# Electronic and Optical Properties of Graphene Oxide Quantum Dots: DFT Insights of Mixed Vacancy and Dopants

**DOI:** 10.1021/acsomega.6c01895

**Published:** 2026-06-22

**Authors:** Edna da Silva Machado, Nailton Martins Rodrigues, João Batista Lopes Martins

**Affiliations:** † Instituto de Química, 28127 Universidade de Brasília, 70910-900 Brasília, DF, Brasil; ‡ Departamento de Química, 37892 Universidade Federal do Maranhão, 65085-580 São Luís, MA, Brasil

## Abstract

In this study, density functional theory (DFT) and time-dependent DFT (TD-DFT) calculations are used to understand the effects of monovacancy defects and heteroatom doping (O, B, N, and P) on the structural, electronic, and optical properties of graphene oxide quantum dots (GOQDs). Structurally, a single vacancy breaks three sp^2^ bonds, while heteroatom substitution at the vacancy site promotes further structural relaxation, most notably in phosphorus-doped GOQDs. DFT-based chemical stability descriptors indicate that pristine GOQD possesses the highest hardness and lowest electrophilicity. Among heteroatom-doped GOQDs, O–N_2_-SV-GOQD has improved electronic stability with high hardness and relatively low electrophilicity. In addition, TDOS and PDOS analyses show that vacancy defects and heteroatom doping significantly modify the electronic structure of GOQDs by introducing new states near the Fermi level and altering the band gap, which can influence their optical response. TD-DFT calculations have been performed to study the optical absorption spectra of different GOQDs. Our results show that vacancy defects and heteroatom doping modify the GOQD spectra, varying their intensity and shifting the bands across the visible region. The LHE results further confirm that vacancy defects and heteroatom doping can affect the light-harvesting performance of GOQDs.

## Graphical abstract

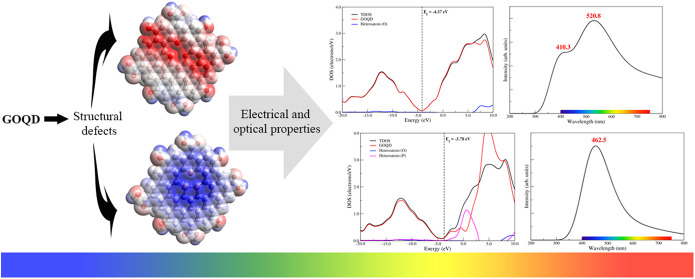

## 1 Introduction

Graphene quantum dots (GQDs) are zero-dimensional nanomaterials with dimensions below 30 nm and thicknesses of only a few atomic layers.^1,2^ GQDs have unique properties that make them useful in photostable materials.^2–6^ These properties include adjustable bandgap, and high biocompatibility. To take advantage of graphene’s properties, it is often necessary to apply external functionalization. The presence of various oxygen-containing groups on the basal plane or edges of the graphene surface yields graphene oxide (GO), which is produced via oxidative methods.

Another finite-size nanomaterial with oxygenated surface groups is graphene oxide quantum dots (GOQDs). Due to their edge effects, high charge density, and quantum confinement, they exhibit improved properties compared to their precursors. These properties have established GOQDs as a promising material for a wide range of applications,^2^ including bioimaging,^7^ drug delivery,^2^ photocatalysts,^8^ energy storage,^9^ and sensors/biosensors.^10–12^ Nonetheless, during GQD/GOQD synthesis, it is virtually unavoidable that structural defects arise within the material, which may compromise not only its structural and morphological integrity but also a broad range of physicochemical properties.^13,14^ Among such defects, the creation of carbon vacancies or chemical doping can introduce new electronic states, alter the band gap, and affect the optical and electrical properties of these quantum dots.^15^

Single or multiple vacancies in graphene materials have been extensively investigated through both experimental and theoretical approaches.^14,16–24^ Mapasha and collaborators investigated, using DFT calculations, the electronic structure of various neutral and charged vacancies in a diamond-like bilayer graphene. They found that charge-state modulation alters the electronic character of the vacancies and suppresses the induced magnetic moment.^23^ Recently, Kuwait and collaborators conducted a theoretical study at the M06–2*X*/6–31G­(d) level to investigate the molecular structures and electronic characteristics of 4N-divacancy-defected graphene quantum dots (4N-GQDs) and metal/metal-ion-doped 4N-GQDs (M–4N-GQDs, where M = Ca, Ca^2+^, Cr, Cr^2+^, Fe, and Fe^2+^) under vertical electric fields. Induced curvature in metal and divalent metal ion-doped 4N-GQDs was reported, altering their electronic properties upon application of an external electric field.^24^ Another practical approach to enhance the electrical and optical properties of these materials involves doping their surfaces with a heteroatom.^5,24–28^ DFT calculations of two graphene oxide nanosheet isomers (GON1 and GON2) and their Al-doped derivatives (GON1-Al1, GON1-Al2, GON1-Al4, GON2-Al1, GON2-Al2, and GON2-Al4) were reported.^29^ They showed that the GON1-Al6 and GON2-Al6 configurations significantly increased the NLO (nonlinear optical) parameter values of the graphene oxide nanosheet. Subsequently, analyzing the optical spectra of the different compounds, the authors observed that GON1-Al1, the GON1 derivative isomer, exhibits the strongest absorption in the visible range.

We aim to address a significant gap in the literature regarding codoping in the presence of a single vacancy. This defect configuration has not been widely explored in the literature on graphene oxide quantum dots (GOQD). Most previous studies^18,19,22,27,30^ focus predominantly on single dopant systems associated with single vacancies or on isolated defects (oxygen groups, vacancies, or single dopants) treated independently. We studied how different dopants present in the vacancy defect can alter its various physicochemical properties. Furthermore, the present work points out this important question by examining the electronic and optical responses of codoped GOQDs containing a single vacancy. This configuration has received limited attention in previous studies. Introducing two chemically distinct dopants into a vacancy site results in subtle but meaningful deviations from the behavior observed in single-doped or single-defect systems. In this context, the purpose is to systematically explore the electronic structure and optical response of vacancy- and heteroatom-doped graphene oxide quantum dots using DFT and TD-DFT calculations at the B3LYP/6–311+G­(d,p) level.

## 2 Computational Details

Quantum chemical calculations were performed at the Density Functional Theory (DFT), utilizing the Gaussian16 software suite.^31^ The hybrid B3LYP functional^32^ was employed with the 6–311+G­(d,p) basis set.^33^ The B3LYP hybrid functional was employed in this work due to its reliability in describing the geometrical and electronic properties and its high accuracy in predicting the optical absorption properties of graphene quantum dots.^34–37^ In addition, a Pople-type basis set was selected due to its well-established track record in previous studies, which have yielded results that closely reproduce experimental observations.^38–41^

Recent studies support the use of B3LYP for systems with preserved sp^2^ domains and moderate oxygen functionalization, conditions that closely resemble those of realistic GO and rGO materials. Recently, TD-DFT/B3LYP calculations^42^ showed very good agreement with experimental UV–Vis spectra of laser-ablated rGO, particularly for π→π* excitations localized in aromatic regions. Similarly, studies of rGO with varying degrees of reduction, where conventional hybrid functionals provided accurate descriptions of local electronic transitions dominated by sp^2^ domains.^43^

In addition, B3LYP was used to evaluate electronic and optical responses of GO nanostructures under solvent effects,^44^ demonstrating that this functional reproduces trends associated with structural changes and environmental interactions.

In our case, the GOQD model preserves conjugated regions in the core, whereas oxygen functionalities are mainly located at the edges. This structure leads to excitations localized within sp^2^ domains, for which B3LYP is known to perform well.^45,46^ Although long-range-corrected functionals may offer improvements, they suffer from tuning the range-separation parameter and also require more computational resources. Furthermore, hybrid functionals such as B3LYP reproduce structural trends and relative spectral variations consistently across chemically modified GQDs and GOQDs, as illustrated in additional works.^47,48^

The convergence of each optimized structure was established by vibrational frequency analysis, in which the absence of imaginary frequencies confirms that the structure is a local minimum on the potential energy surface. For the excitation transitions of GOQDs, the 30 lowest excited states were calculated using the time-dependent density functional theory (TD-DFT) method.^49^ The absorption spectra were fitted with a Gaussian function with a half-width at half-maximum (HWHM) of 0.13 eV.

Frontier molecular orbital analysis was used to analyze the effects of different defects on the electronic structure and chemical reactivity of GOQD. Calculation of chemical descriptors, such as ionization potential and chemical hardness, has been widely explored in the literature concerning the impact of different methods.^50–52^ Specifically, using the DFT formalism, the vertical ionization potential (VIP) and the vertical electron affinity (VEA) were determined from the following Equations^53^

1 $$VIP={E\left(GOQD\right)}^{+}-E\left(GOQD\right)$$

2 $$VEA=E\left(GOQD\right)-{E\left(GOQD\right)}^{-}$$

where *E*(GOQD) is the total energy of the neutral GOQD at its optimized geometry, *E*(GOQD)^−^ corresponds to the energy of the anionic state evaluated at the neutral geometry, and *E*(GOQD)^+^ is the corresponding energy of the cationic state computed without allowing structural relaxation.^54^

From the VIP and VEA values, the chemical potential (μ) and chemical hardness (η) were determined.^53^ The following equations express these parameters

3 $$\eta =\frac{1}{2}\left(\mathrm{VIP}-\mathrm{VEA}\right)$$

4 $$\mu =\frac{-1}{2}\left(\mathrm{VIP}+\mathrm{VEA}\right)$$

Parr and co-workers^55^ introduced the electrophilicity index (ω) based on the chemical potential and chemical hardness.

5 $$\omega =\frac{\mu^{2}}{2\eta }$$

Visualization of structural geometry, molecular orbitals, and molecular electrostatic potential (MEP) maps is performed using Avogadro software.^56^

## 3 Results and Discussion

### 3.1 Structural and Stability Properties

To investigate the effects of defect types on GOQDs, we used C_58_H_18_O_12_ as a GOQD model (see Figure 1) and introduced two defect classes: vacancy defects and heteroatom doping (O, B, N, and P). GOQD models with hexagonal geometries have been widely explored in the literature,^19,22,27,57^ supporting this structural motif.

**Figure 1:**
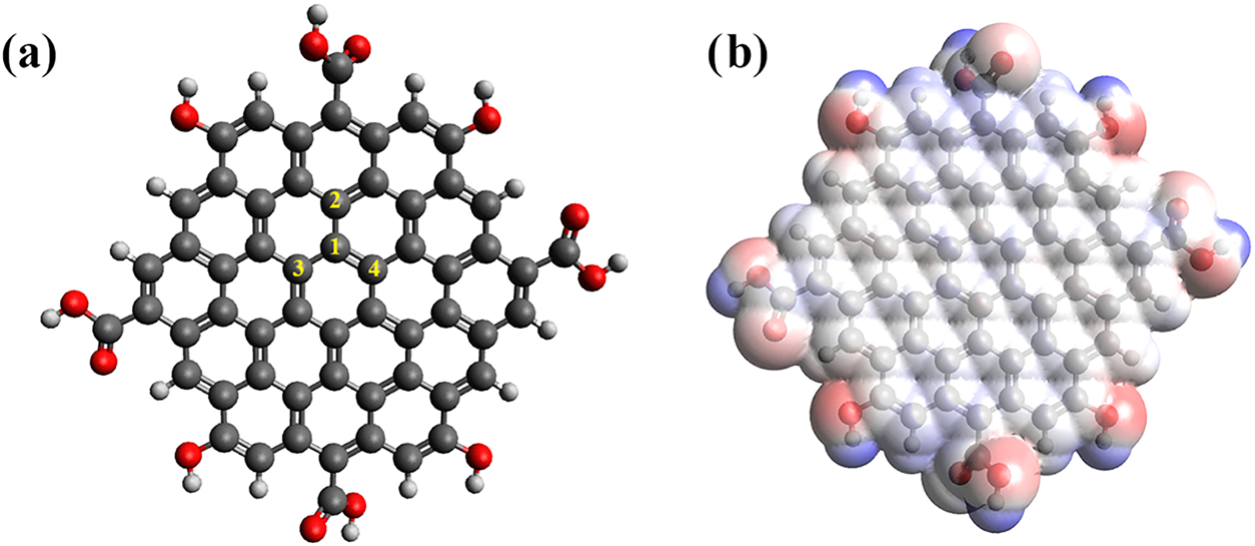
(a) Optimized structure, and (b) Molecular electrostatic potential (MEP) maps across a surface of total electron density for the pristine GOQD model. Highlighted atoms indicate the removed site (C_1_) and the substitution sites (C_2_ to C_4_) used for introducing different heteroatoms in the GOQD structures. In MEP, the blue color corresponds to regions with excess positive charge, the red color represents the negatively charged regions, and the gray color to regions with neutral or near-zero electrostatic potential.

Initially, a vacancy was created by removing a carbon atom from the basal plane of the structure (atom C1), resulting in a single vacancy (SV) referred to as SV-GOQD. Subsequently, to form the structures with the doping atoms, it was necessary to maintain the same spin multiplicity (singlet), all structures are closed shell. The first vacancy-doped GOQD was obtained by substituting a carbon atom (C2) with oxygen, and this quantum dot is designated as O–SV-GOQD. For the formation of the other GOQDs, the previously inserted oxygen atom was kept fixed, and the other carbon atoms (C3–C4) were replaced by different heteroatoms (O, B, N, and P). The following designations have been assigned to these structures: O_3_–SV-GOQD, O–B_2_-SV-GOQD, O–N_2_-SV-GOQD, and O–P_2_-SV-GOQD. Figure S1 (Supporting Information) summarizes this information. The optimized ground state geometries and molecular electrostatic potential (MEP) surfaces of GOQDs with defects are shown in Figure 2.

**Figure 2:**
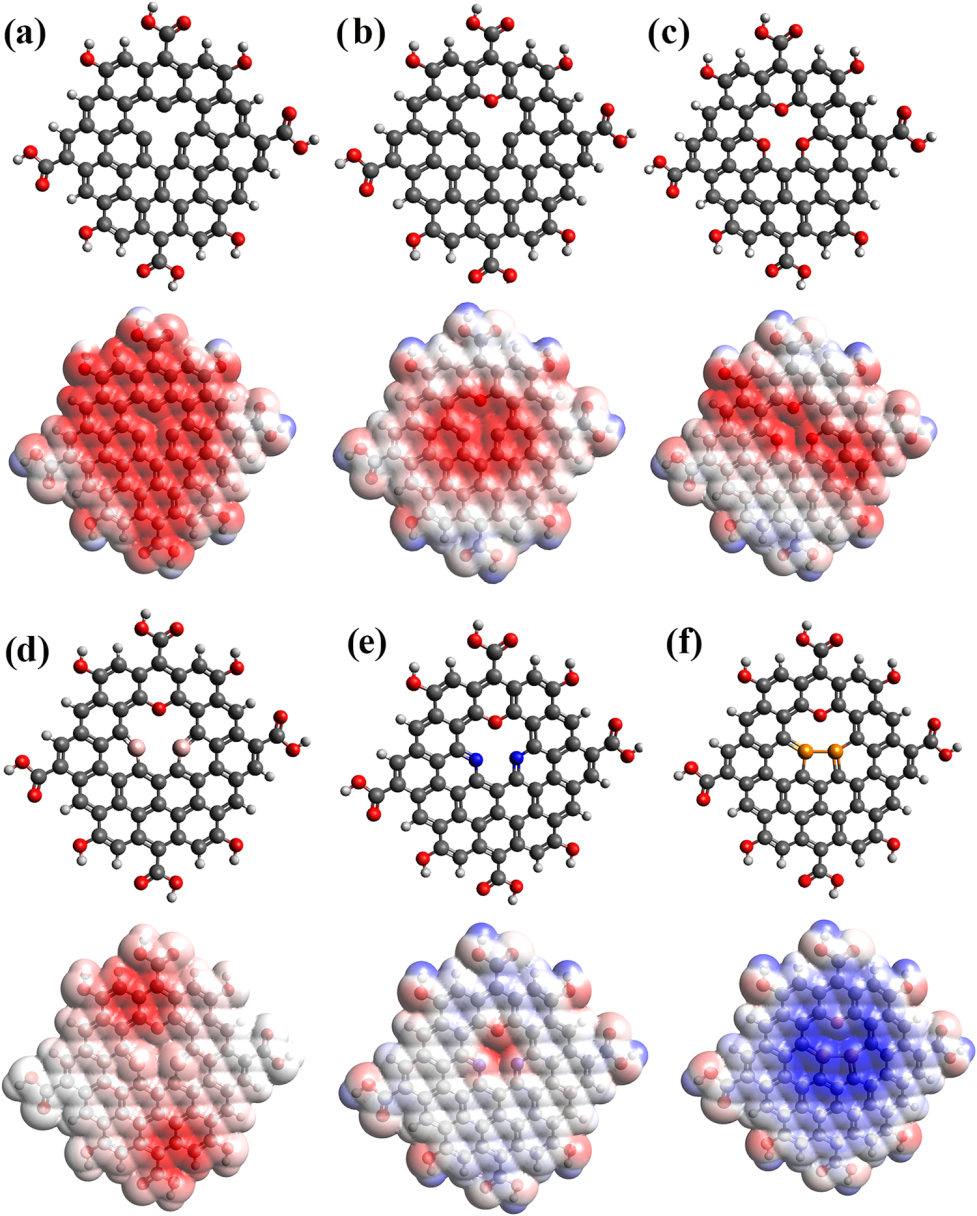
Optimized structure and its respective MEP graphic analysis of (a) SV-GOQD, (b) O–SV-GOQD, (c) O_3_–SV-GOQD, (d) O–B_2_-SV-GOQD, (e) O–N_2_-SV-GOQD, and (f) O–P_2_-SV-GOQD. In MEP, the blue color corresponds to excess positive charge, the red color represents negatively charged regions, and the gray color generally to regions with neutral or near-zero electrostatic potential.

The generated single-vacancy defect (Figure 2a) has three sp^2^ bonds broken. Consequently, the electrons associated with these missing bonds tend to be localized in the vicinity of the vacancy site. When comparing the bond lengths (Supporting Information) before and after the removal of a carbon atom, no significant changes were observed in the GOQD skeleton (Figure S1). The introduction of various heteroatoms (O, B, N, and P) induces geometric and electronic changes, with the phosphorus-doped GOQD exhibiting the largest structural relaxation and charge redistribution (Table S1).

Analysis of the MEP surfaces for the different GOQDs shows variations in their electrostatic distributions. MEP red areas correspond to highly negative potential, indicative of electrophilic behavior, whereas blue areas are the highly positive potential, associated with nucleophilic activity. As shown by the MEP in Figure 1, pristine GOQD is largely neutral, with only localized deviations at unsaturated carbon sites along the edges. Therefore, pristine GOQD offers a limited number of accessible adsorption sites, thereby constraining its potential for surface interactions. In contrast, the analysis of MEPs in the presence of structural defects presents substantial variations in electrostatic distribution (Figure 2). The introduction of a single vacancy in GOQD (SV-GOQD) significantly modifies the surface electrostatic potential, as illustrated in Figure 2a. The removal of a carbon atom produces a distinct red region in the electrostatic potential map, indicating an electron-rich zone distributed across the entire surface with increased negative charge concentration at and around the vacancy.^58–60^

On the other hand, the incorporation of heteroatoms (O, B, N, and P), as can be seen in the Figure 2b–f, into GOQD induces significant modifications in the electronic distribution, with the extent and nature of these changes governed by the specific heteroatom introduced. As shown in Figure 2b, the MEP surface of the structure containing a central oxygen atom and an associated vacancy (O-GOQD) displays a negative electrostatic potential close to the substituted oxygen, reflecting enhanced electron density at the defect area. In contrast, positive electrostatic potential is primarily observed in the peripheral regions, reflecting a vacancy-induced radial redistribution of charge.^61^ For the O_3_–SV-GOQD (Figure 2c), the MEP analysis shows that the vacancy coordinated by three oxygen atoms promotes partial charge delocalization, leading to a redistribution of the electrostatic potential toward the hydroxyl (OH) groups.^62^ Moreover, compared to the O_3_ molecule, the distance of O–O is 2.577 Å, which is larger than the attributed experimental value of 1.272 Å for the O_3_ molecule. The MEP of the O–B_2_-SV-GOQD (Figure 2d) exhibits intense red regions mainly distributed along the peripheral areas, indicating electron-rich domains associated with delocalized π systems.^63^ The MEP of the O–N_2_–SV-GOQD (Figure 2e) exhibits a pronounced negative electrostatic potential localized at the center of the quantum dot, primarily associated with the oxygen atom occupying the vacancy. The adjacent nitrogen atoms function as compensating centers, modulating the local charge distribution and generating a strongly polarized defect region, indicative of enhanced charge localization and reduced π-electron delocalization.^64^

The replacement of carbon atoms with phosphorus led to the formation of a P–P covalent bond with an equilibrium bond length of 2.105 Å, as determined from the optimized geometry, which is consistent with previously reported P–P bond lengths, typically around 2.22 Å.^65–67^ The MEP of the O–P_2_–SV-GOQD (Figure 2f) is predominantly positive, with extensive blue regions distributed over the central area of the quantum dot. Compared to the O–P_2_-SV-GOQD, which has a bond distance of P–P equal to 2.105 Å, the P_2_ molecule has a bond distance of 1.895 Å, and in the P_4_ is 2.223 Å,^68^ while the calculated BP86/TZ2P is 1.911 for P_2_.^69^ This difference arises from the distinct chemical environment phosphorus occasions upon incorporation into the GOQD lattice. In the free P_2_ molecule, the P–P bond exhibits a strong multiple-bond character, which accounts for its short equilibrium bond length.^70,71^ In the O–P_2_–SV–GOQD configuration, each phosphorus atom simultaneously interacts with neighboring oxygen atoms at the vacancy site. These additional P–C and P–O interactions disrupt the host lattice’s π-conjugated network and compete with P–P bonding, effectively reducing the P–P bond order compared to an isolated P_2_ molecule.^72^ Although the P–P bond in the doped system retains its covalent character, charge redistribution and partial electron delocalization toward the substrate lead to a noticeable bond elongation.^73^

This behavior is attributed to the incorporation of phosphorus atoms at the vacancy site, which breaks the π-conjugated network and weakens P_2_ bonding, leading to a slightly larger bond distance than in the P_2_ molecule and thereby inducing electronic depletion and an overall positive electrostatic potential.^74–76^ Phosphorus substitution is the only one that presents the orbital overlap between the bonding of P_2_, as shown in the HOMO orbital (Figure S2). Although the oxygen atom contributes to a localized negative region, its effect is insufficient to counterbalance the global charge depletion imposed by the phosphorus atoms, resulting in a net positive electrostatic potential.

Quantum chemical stability descriptors, such as the chemical potential (μ), global hardness (η), and electrophilicity index (ω), play a crucial role in predicting chemical stability and elucidating the reactivity of molecular systems.^77^ Figure 3 presents the values of these descriptors for the different GOQDs investigated, determined using eqs 1–5.

**Figure 3:**
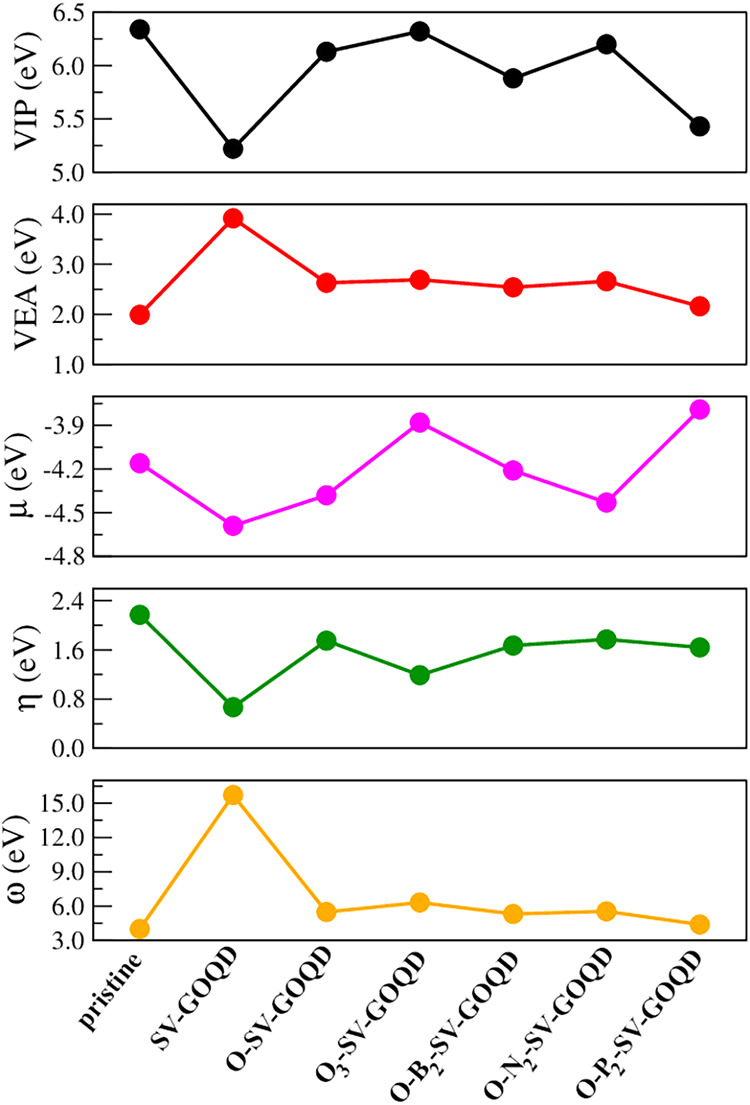
Reactivity descriptors for GOQDs.

The VIP values indicate that pristine GOQD exhibits the highest ionization potential (6.34 eV), suggesting more strongly bound electrons and greater electronic stability.^78^ In contrast, the reduced VIP observed for SV-GOQD (5.22 eV) suggests that vacancy defects weaken electron binding, thereby increasing the trend for electron donation and chemical reactivity. This result is consistent with previous DFT studies demonstrating that vacancy defects in graphene quantum dots introduce localized electronic states and weaken electron binding, thereby lowering the ionization energy.^3,79^

The uniformly positive VEA values observed for all GOQDs indicate that electron addition is energetically favorable, reflecting a pronounced electron-accepting ability in these systems. Figure 3 indicates that all defect-containing GOQDs have higher VEA values than the pristine GOQD, particularly SV-GOQD, which shows the largest VEA (3.92 eV).

The principal global reactivity descriptors (μ, η, and ω) were derived from the VIP and VEA values according to eqs 3–5 (Figure 3), leading to differences in the chemical potential between pristine and defective GOQDs. The chemical potential decreases upon vacancy introduction, from −4.16 eV in pristine GOQDs to −4.59 eV in SV-GOQD, suggesting enhanced electronic stability of the defective structure. A similar trend is observed for vacancy doping with different heteroatoms, as evidenced by O–SV-GOQDs (−4.38 eV), O–B_2_-SV-GOQDs (−4.21 eV), and O–N_2_-SV-GOQDs (−4.43 eV), which all exhibit lower chemical potentials than defect-free GOQDs, indicating an enhanced tendency to accept electrons. In contrast, O_3_–SV-GOQD (−3.88 eV) and O–P_2_-SV-GOQD (−3.79 eV) display higher chemical potentials, suggesting a reduction in electronic stability.

Chemical hardness (η) reflects a system’s resistance to electronic redistribution, whereas the electrophilicity index (ω) measures its tendency to accept electronic charge. According to the maximum hardness principle and the minimum electrophilicity principle, chemically stable systems are commonly associated with higher η and lower ω values.^55,80–83^ For the GOQDs investigated, the chemical hardness decreases in the following order: pristine GOQD (2.17 eV) > O–N_2_-SV-GOQD (1.77 eV) > O–SV-GOQD (1.75 eV) > O–B_2_-SV-GOQD (1.67 eV) > O–P_2_-SVGOQD (1.64 eV) > O_3_–SV-GOQD (1.19 eV) > SV-GOQD (0.67 eV). This trend indicates that the pristine GOQD exhibits the highest chemical stability, whereas the introduction of vacancies and heteroatom doping progressively reduces hardness, suggesting enhanced chemical reactivity.^80^ This reduction in hardness is consistent with experimental observations showing that defect-rich graphene oxide structures are more susceptible to oxidation, adsorption processes, and structural degradation under ambient conditions, as reported in previous studies.^3,42,43,84^

Lower ω values indicate reduced electrophilic character and enhanced electronic stability. On this basis, the electrophilicity of the GOQDs increases in the order: pristine GOQD (4.00 eV) < O–P_2_-SV-GOQD (4.39 eV) < O–B_2_-SV-GOQD (5.31 eV) < O–SV-GOQD (5.48 eV) < O–N_2_-SV-GOQD (5.54 eV) < O_3_–SV-GOQD (6.32 eV) < SV-GOQD (15.72 eV), highlighting the strong electrophilic nature of the vacancy-containing structure. The phosphorus-doped system, however, slightly deviates from this trend, as its relatively higher chemical potential moderates the increase in ω. The high electrophilicity index obtained for SV-GOQD is consistent with theoretical studies showing that vacancy defects in graphene and graphene oxide significantly increase the electron-acceptor character due to increased electronegativity and reduced hardness. It is known that graphene sheets rich in defects and fragmented sp^2^ domains generate localized acceptor states. Furthermore, theoretical studies on graphene quantum dots report electrophilicity values comparable to those obtained here, indicating that strong electrophilic behavior is an inherent feature of vacancy-containing carbon quantum dots.^14,79,85^

### 3.2 Electronic Properties Analysis

Density of states (DOS) analysis aids the study of the electronic structure of GOQDs, in the analysis of how defects alter the distribution of electronic states. The total and projected densities of states (TDOS and PDOS) are shown in Figure 4.

**Figure 4:**
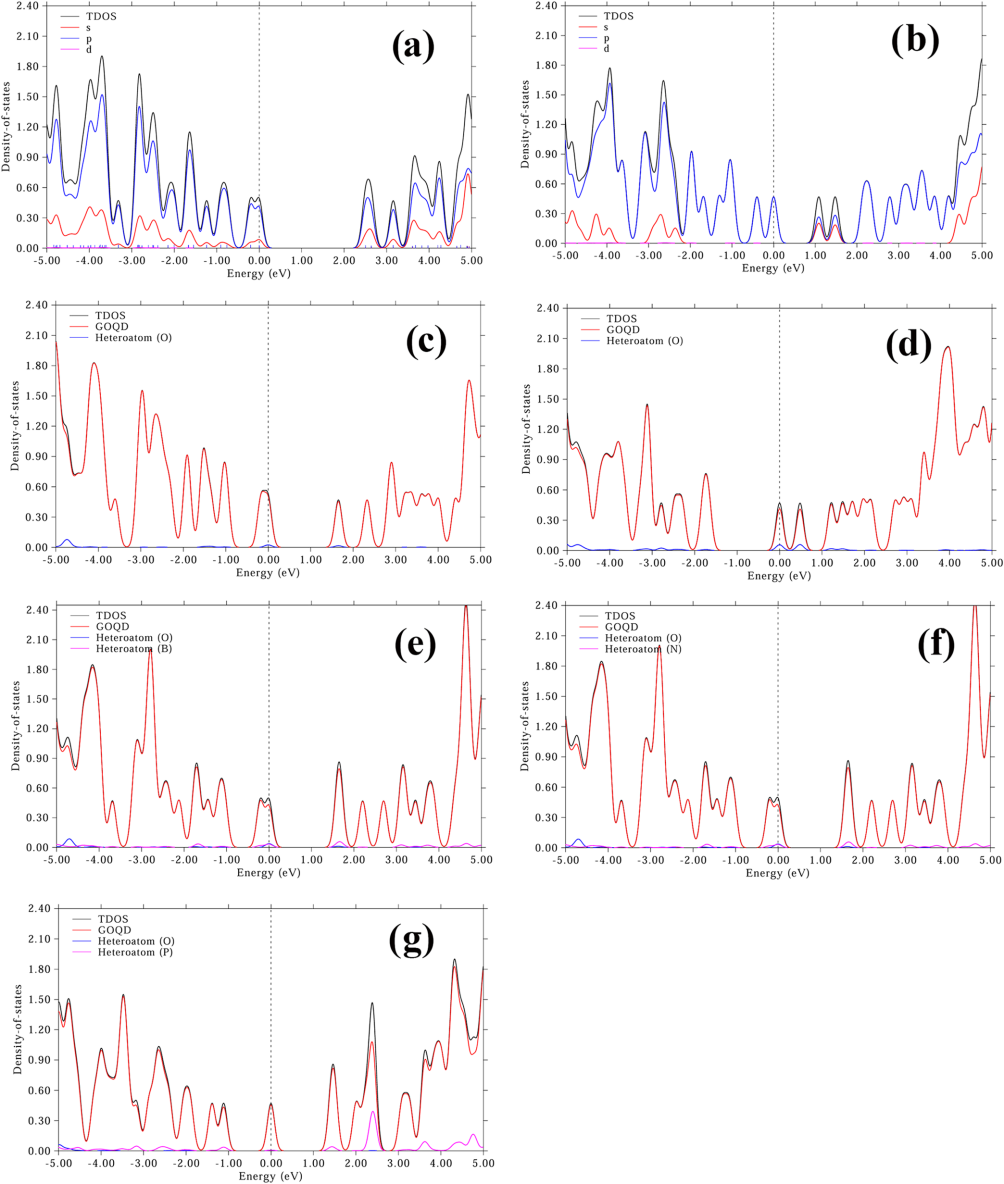
Total and projected densities of states (TDOS and PDOS) plots of (a) pristine GOQD, (b) SV-GOQD, (c) O–SV-GOQD, (d) O_3_–SV-GOQD, (e) O–B_2_-SV-GOQD, (f) O–N_2_-SV-GOQD, and (g) O–P_2_-SV-GOQD. The dashed line at 0 eV indicates the HOMO-aligned energy reference.

We first compare the electronic structures of the pristine GOQD and the GOQD containing a single vacancy (SV-GOQD) through their density of states (DOS) and frontier orbital energies (Figure S2). Pristine GOQDs exhibit a band gap (*E*_g_) of approximately 2.50 eV, with no states observed at the Fermi level (*E*_f_ = −4.15 eV), characterizing them as semiconductor-like materials whose band-edge states are dominated by π and π*, derived p-orbital contributions. Compared to pristine GOQD, introducing a single vacancy (SV-GOQD) lowers the Fermi level to −4.45 eV. The valence states have no significant change, while conduction states have changes mostly in the intensity, particularly those associated with the s and p orbital contributions. Several studies on graphene quantum dots show that vacancy formation disrupts the π-conjugated network and redistributes electron density near the Fermi level, naturally shifting its energy position.^79,86–88^

The introduction of different heteroatom combinations into the defective SV-GOQD results in distinct modifications to the electronic structure, particularly the position of the Fermi level and the distribution of states near the valence and conduction bands (Figure 4c–g). Likewise, the most notable change is in the conduction band. Boron and phosphorus also show certain similarities in the MEP of the edge of these clusters. The O–N_2_–SV-GOQD (Figure 4f) exhibits the most negative Fermi level among the quantum dots examined. This result is similar to studies showing that incorporating nitrogen near vacancy sites in graphene significantly disrupts the electronic structure and introduces impurity levels close to the Fermi energy^89^ For the O–P_2_–SV-GOQD, the Fermi level increases to – 3.78 eV, and pronounced phosphorus-derived states appear in the low-energy portion of the conduction band (−1 to 3 eV). This feature is characteristic of donor-type behavior near vacancy sites.^90,91^

### 3.3 Optical Properties of GOQDs

TD-DFT calculations were performed to investigate the absorption spectra of GOQDs with vacancy defects and various heteroatom dopants. Figure 5 presents the absorption spectra.

**Figure 5:**
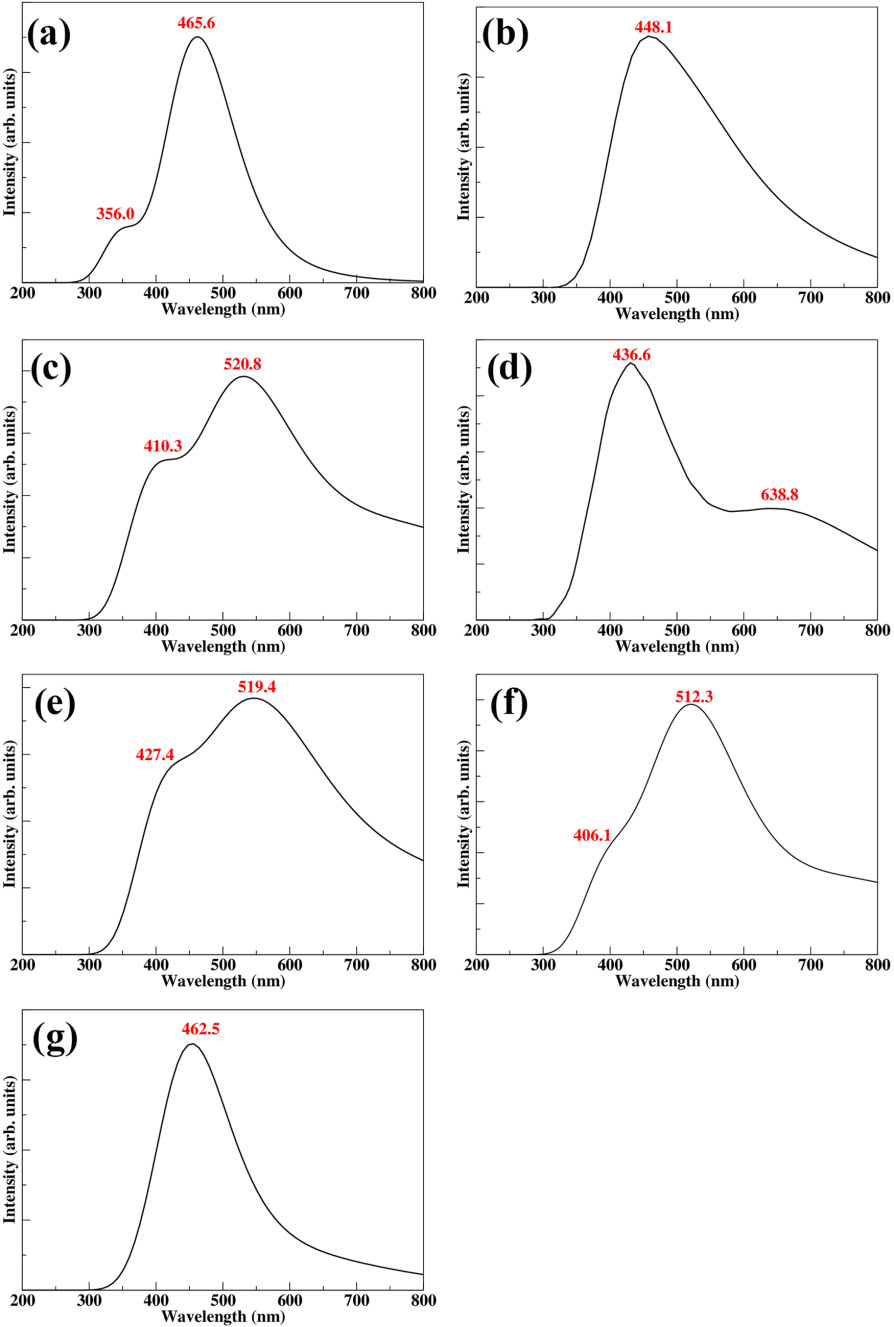
Optical absorption spectra of (a) pristine GOQD, (b) SV-GOQD, (c) O–SV-GOQD, (d) O_3_–SV-GOQD, (e) O–B_2_-SV-GOQD, (f) O–N_2_-SV-GOQD, and (g) O–P_2_-SV-GOQD.

Analysis of the absorption spectrum of pristine GOQD (Figure 5a) shows two characteristic peaks at 356.0 nm (oscillator strength, *f* = 0.106) and 456.0 nm (*f* = 0.895). The higher-energy peak is primarily associated with the HOMO–2 → LUMO+2 electronic transition. The low-energy, more intense peak at 456.0 nm is primarily dominated by the HOMO–1→LUMO+1 transition, with a nearly comparable contribution from the HOMO → LUMO+1 transition, indicating the mixed character of the electronic excitations. Both transitions are predominantly of π → π* character, arising from the conjugated sp^2^ carbon framework of pristine GOQD. The presence of a single vacancy (SV-GOQD, Figure 5b) shifts the highest-intensity peak to 448.1 nm (*f* = 0.415), indicating a blueshift relative to the pristine structure. This peak is predominantly associated with the HOMO–2 → LUMO+2 electronic excitation, which is the primary contributor to the observed spectral feature.

In order to determine the character of these transitions, we have calculated the charge-transfer (CT) Δ*r* index and the total CT excitation length (*D*) index.^92,93^ Table 1 shows the *r* and *D* indices, and these values indicate a major CT character for the O_3_–SV-GOQD, O–B_2_-SV-GOQD, and O–P_2_-SV-GOQD. In the case of a conjugated organic aromatic, it was reported that a ground state transition of Δ*r* > 2.0 can be considered as a CT-type transition.^92^ Graphene and graphene oxide QDs have a higher density of π-electrons; as such, Δ*r* is a good qualitative descriptor for the CT nature of a transition.^27^ These CT have π→π* character, as described by the molecular orbitals involved in the transitions (Figure S3).

**Table 1:** Charge Transfer Length Δ*r* (Å) and the Total Length *D* (Å) Index for the Quantum Dots Compared with the Pristine GOQD

	Δ*r*	*D*
pristine GOQD	0.0013	0.002
SV-GOQD	1.6148	1.377
O–SV-GOQD	1.2938	1.729
O_3_-SV-GOQD	2.8210	3.158
O–B_2_-SV-GOQD	2.1520	0.864
O–N_2_-SV-GOQD	0.6327	0.963
O–P_2_-SV-GOQD	2.3130	2.102

As shown in Figure 6, orbital analysis indicates that HOMO–2 is primarily composed of π-type C­(2p) orbitals, while LUMO+1 exhibits mixed contributions from C­(2p) and C­(2s) orbitals induced by the defect. Despite this orbital hybridization, the electronic excitation have π → π* contribution. The shift toward higher energy regions observed in the π → π* transition of SV-GOQD is directly related to the disruption of the sp^2^ conjugated lattice caused by the formation of single vacancies. This disruption locally fragments the aromatic domains and reduces the delocalization of π orbitals. Consequently, the increased orbital localization raises the energy gap between the π and π* states, leading to higher excitation energies compared to pristine GOQD. Similar trends have been documented in defect-rich graphene oxide systems, in which disruption of sp^2^ conjugation reduces π-orbital delocalization, thereby increasing the π–π* transition energy and producing a blueshift in the absorption spectrum.^14,43^

**Figure 6:**
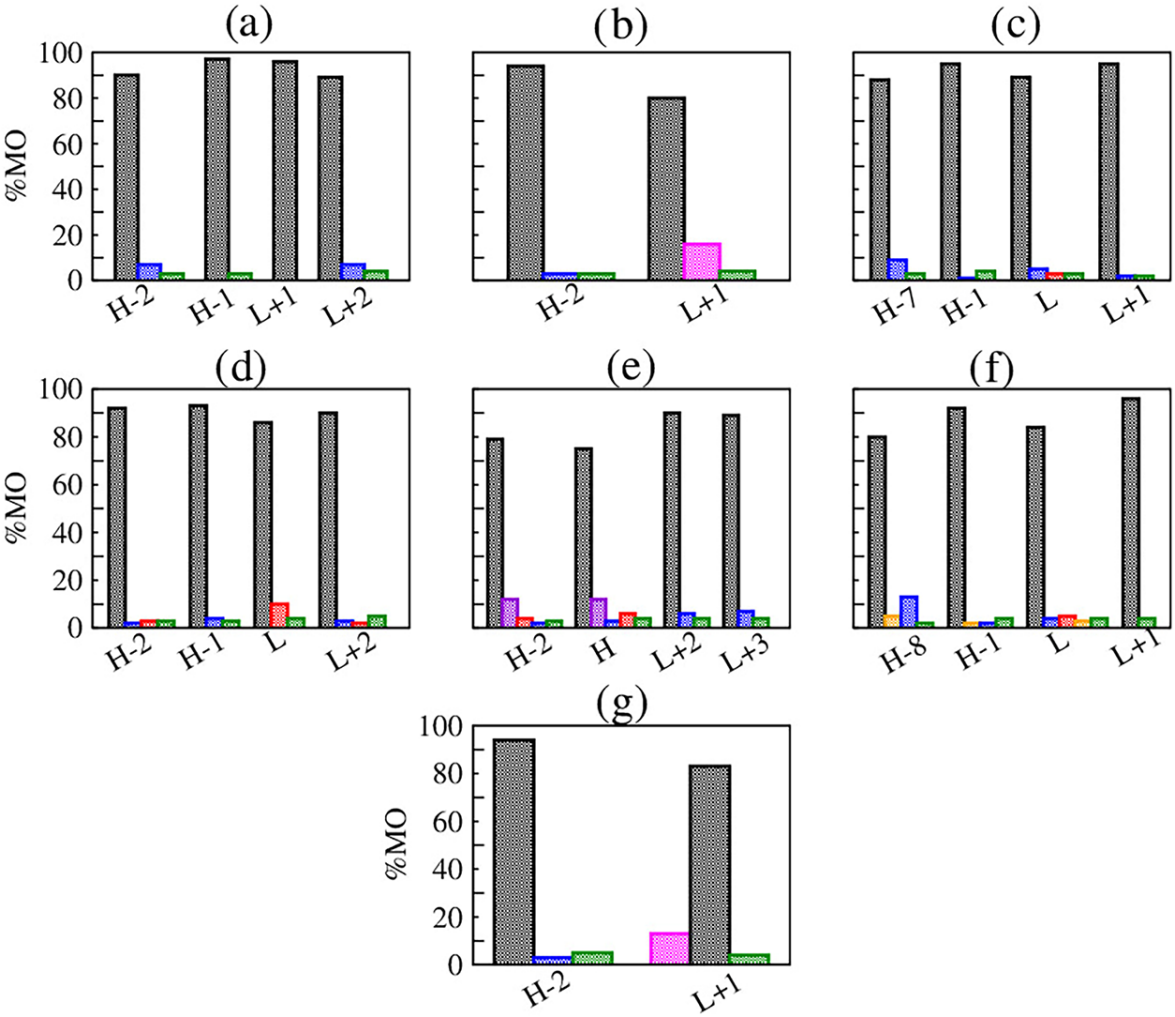
Graphical representation of the contribution of atomic orbitals in the formation of the molecular orbitals involved in the electronic transitions of the (a) pristine GOQD, (b) SV-GOQD, (c) O–SV-GOQD, (d) O_3_–SV-GOQD, (e) O–B_2_-SV-GOQD, (f) O–N_2_-SV-GOQD, and (g) O–P_2_-SV-GOQD. The bar colors represent contributions from different atomic orbitals. Black corresponds to C­(2p), magenta to C­(2s), blue to O­(2s), red to the O­(2p) orbitals of the heteroatom, violet to B­(2p), orange to N­(2p), and green to the d orbitals. * Detailed compositions of orbitals with contributions below 20% are presented in Figure S4.

Analysis of the O–SV-GOQD absorption spectrum (Figure 5c) shows two peaks at 410.3 nm (which are mainly composed of transitions HOMO–7 → LUMO and HOMO → LUMO+4) and 520.8 nm (attributed mainly to the electronic transition HOMO–1 → LUMO+1), with oscillator strengths of 0.288 and 0.667, respectively. Although the overall spectral profile is similar to that of the pristine structure, the highest intense peak shifts toward longer wavelengths, indicating a redshift. All the molecular orbitals involved in the electronic transitions mentioned above have a predominant carbon 2p character, with contributions above 85% (Figure 6). Therefore, both excitations can be classified as π → π* transitions. The broad shoulder observed at 520.8 nm in O–SV-GOQD is attributed to oxygen-related excited states. These states occur within the same spectral region as the HOMO–1 to LUMO+1 transition. The excitations have similar oscillator strengths and partially overlap, leading to a broadened combined feature. In contrast, the pristine structure is more symmetric, leading to more isolated excitations and, as a result, a single dominant absorption peak in this spectral region.

The O_3_–SV-GOQD spectrum (Figure 5d) differs from the other GOQDs by displaying its most intense absorption peak at 436.6 nm (*f* = 0.415), along with a broad low-energy peak at 638.4 nm (*f* = 0.208). The strong peak arises mainly from the HOMO–1 → LUMO+2 electronic transition, which is the dominant contribution to this spectral feature. Orbital analysis shows that both orbitals are predominantly composed of C­(2p) states, with only minor contributions from oxygen 2p orbitals (Figure 6). Accordingly, this excitation can be classified as a predominantly π → π* transition. The lowest-intensity peak is predominantly associated with the HOMO–2 → LUMO electronic transition, which is dominated by carbon π states but exhibits noticeable oxygen 2p contributions in the LUMO, indicating a predominantly π → π* transition with mixed orbital character.

Boron-doping effects are reflected in the optical absorption spectrum Figure 5e, which displays two absorption features: a weaker peak at 427.4 nm (*f* = 0.209) and a stronger band at 519.4 nm (*f* = 0.421). The lowest-intensity peak arises mainly from the HOMO–2 → LUMO electronic transition and provides only a minor contribution to the overall absorption profile. The occupied orbital is predominantly composed of C­(2p) orbitals, with additional contributions from heteroatom-derived states, whereas the unoccupied orbital shows negligible heteroatom participation. The transition can therefore be classified as a predominantly π → π* excitation. In contrast, the absorption peak at 519.4 nm is predominantly associated with the HOMO→LUMO+3 transition, with an additional contribution from the HOMO–2→ LUMO transition, in which a more strongly hybridized HOMO is promoted to a largely carbon-based π state, resulting in a lower excitation energy compared to the 427.4 nm peak. PDOS analysis (Figure 4e) shows pronounced B and O contributions near the Fermi level, evidencing a strongly hybridized HOMO. At the same time, deeper states remain carbon-like, explaining the red-shifted π → π* transition at 519.4 nm.

The absorption spectrum of O–N_2_-SV-GOQD (Figure 5f) exhibits a profile similar to that of O–B_2_-SV-GOQD, featuring two characteristic absorption peaks centered at 406.1 and 512.3 nm. The higher-energy absorption band at 406.1 nm (*f* = 0.123) is predominantly associated with the HOMO–8 → LUMO electronic transition, with an additional contribution from the HOMO–6 → LUMO+1 transition. The more intense absorption band at 512.3 nm (*f* = 0.608) arises exclusively from the HOMO–1 → LUMO+1 electronic excitation, consistent with a π → π* transition, as shown in Figure 6. The blue shift observed for O–N_2_-SV-GOQD relative to its boron-doped counterpart arises from the donor nature of nitrogen atoms, which promotes a more uniform hybridization of the frontier orbitals and results in higher-energy electronic excitations.^94^

Finally, the absorption spectrum of O–P_2_-SV-GOQD (Figure 5g) exhibits a peak at 462.5 nm (*f* = 0.612), primarily associated with the HOMO–2 → LUMO+1 transition. The similarity in both the spectral profile and peak position relative to pristine GOQD suggests that phosphorus doping preserves the system’s intrinsic optical response.

To obtain a clearer description of the dominant excited states, Natural Transition Orbitals (NTOs) were computed for the most intense absorption band of each GOQD (Figure 7). NTOs provide a compact and unambiguous representation of the underlying electronic transition by reducing it to the leading hole–particle orbital pair. The resulting NTOs confirm that the dominant excitation in all systems retains a π→π* character, with hole and particle densities primarily distributed over the carbon π framework. It is noted that vacancies in different GOQDs exhibit stronger spatial localization of both hole and particle densities around the defect region, reflecting sp^2^ lattice disruption. In contrast, doped GOQDs exhibit intermediate localization. The corresponding NTO weights (51–80%) indicate that the leading hole–particle pair provides the dominant contribution to the electronic excitation, whereas systems with lower weights show additional, non-negligible contributions from higher NTO pairs, supporting the assignments derived from TD-DFT and PDOS analyses.

**Figure 7:**
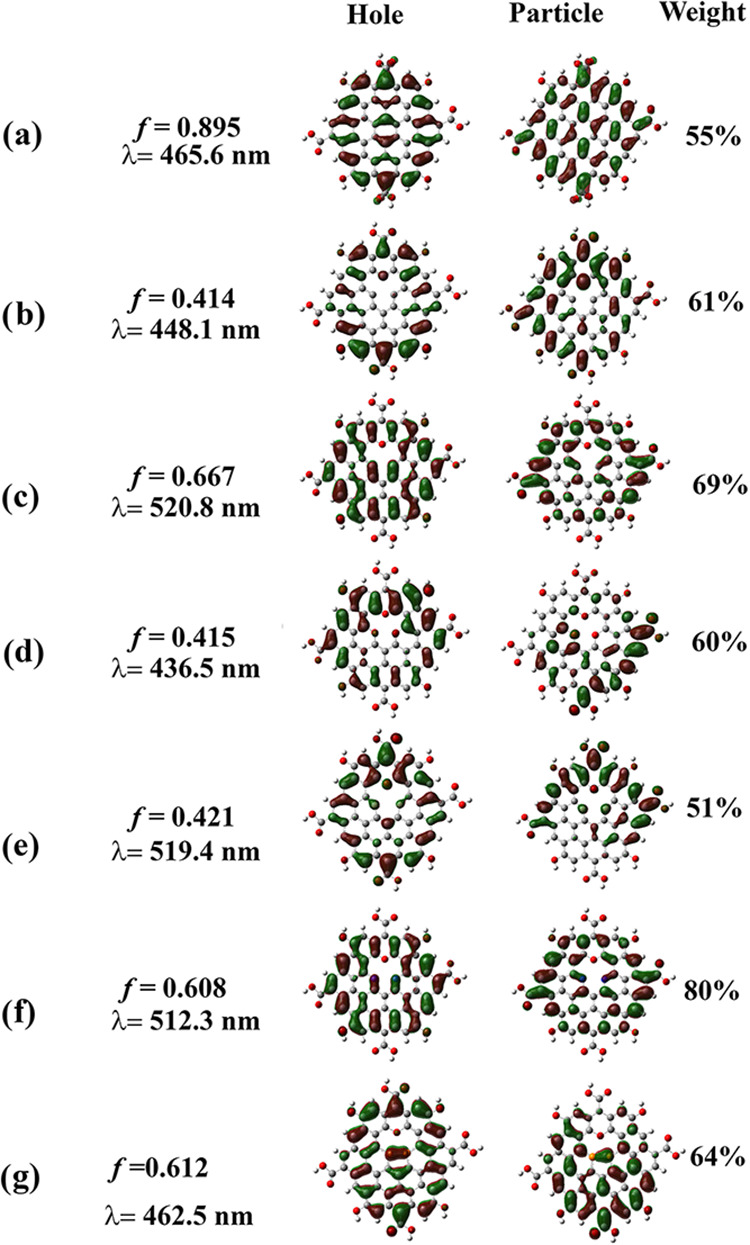
Natural transition orbitals (NTOs) for the dominant electronic excitation associated with the maximum absorption band of (a) pristine GOQD, (b) SV-GOQD, (c) O–SV-GOQD, (d) O_3_–SV-GOQD, (e) O–B_2_-SV-GOQD, (f) O–N_2_-SV-GOQD, and (g) O–P_2_-SV-GOQD. For each system, oscillator strength (*f*), excitation wavelength, and NTO weight (%) are reported.

The optical efficiency of a material can be characterized by the light-harvesting efficiency (LHE), a dimensionless parameter derived from oscillator strengths that quantifies the system’s light-absorption capability, and can be expressed as

6 $$\mathrm{LHE}=1-10^{-f}$$

where *f* corresponds to the oscillator strength of the dominant electronic transition contributing to the maximum absorption band. Figure 8 shows the LHE values obtained for pristine and defective GOQD structures at the wavelength corresponding to maximum oscillator strength.

**Figure 8:**
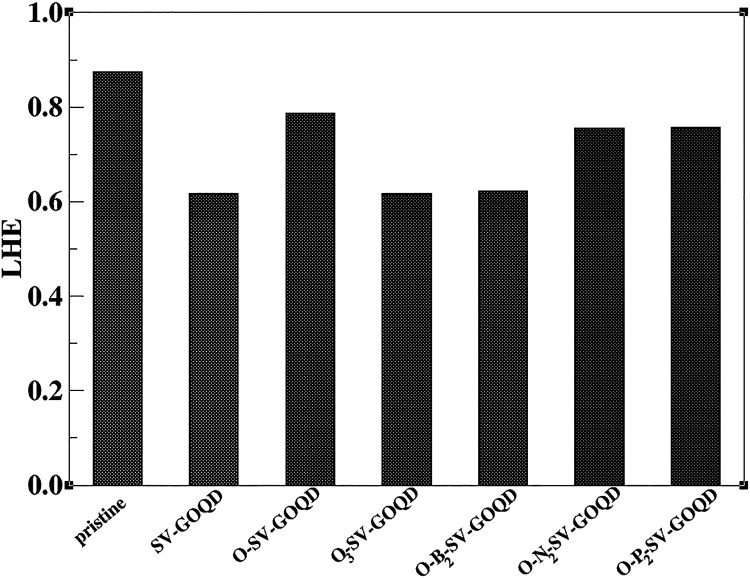
Light harvesting efficiency (LHE) of GOQDs with and without defects computed by the TD-DFT method.

Pristine GOQD exhibits the highest LHE value (0.873), whereas systems containing vacancies or dopants show intermediate values ranging from 0.60 to 0.78. These differences reflect modifications in the energies and oscillator strengths of the dominant electronic transitions predicted by TD-DFT upon defect introduction, rather than a systematic improvement in light-harvesting capability. Because LHE is directly derived from oscillator strengths, it should be interpreted as a qualitative measure of relative optical activity among the studied systems, not as an indicator of device-level performance. For this reason, the discussion is restricted to theoretical trends, without extending the analysis to practical optoelectronic efficiencies.^15,95–97^

## 4 Conclusion

Density functional theory was employed to evaluate the effects of various defects on the structural, electronic, and optical properties of GOQDs. The defects considered include single-vacancy formation in pristine GOQD, yielding SV-GOQD, as well as heteroatom doping, resulting in O–SV-GOQD, O_3_–SV-GOQD, O–B_2_-SV-GOQD, O–N_2_-SV-GOQD, and O–P_2_-SV-GOQD. Based on structural analysis, the planarity of the structure is maintained in the presence of a single vacancy and upon dopant incorporation in the central region of the basal plane. Overall, the molecular electrostatic potential (MEP) map analysis indicates that single-vacancy defects minimally perturb the global electrostatic profile of GOQDs, whereas heteroatom incorporation promotes intense local polarization. This behavior underscores the effectiveness of defect manipulation as a strategy for precise control of electronic structure.

The main global reactivity descriptors (μ, η, and ω) indicate that pristine GOQD is the most chemically stable system. In contrast, SV-GOQD and heteroatom doping increase reactivity, reducing hardness and increasing electrophilicity. Among the doped GOQDs, O–N_2_-SV-GOQD was the most electronically stable.

We also investigated the density of states (DOS) for the different GOQDs. The DOS results show that heteroatom doping at the vacancy site enables fine-tuning of GOQD electronic properties by modulating charge redistribution and the density of states.

TD-DFT calculations suggested that vacancy defects and heteroatom doping in GOQDs induce pronounced red and blue shifts in the optical absorption spectra, highlighting defect-driven modulation of the electronic structure as a strategy for tuning GOQD optical properties. The LHE values illustrate how the introduction of defects redistributes oscillator strengths among low-energy excitations. However, they do not indicate a consistent increase in light-harvesting capability. Since LHE is calculated solely from oscillator strengths, it provides a qualitative comparison within the studied series but does not yield conclusive insights into device performance.

## Supplementary Material



## Data Availability

All data and information for the reproduction of these works are available in the text.

## Notes

Schematic route for the generation of defective GOQDs, Mulliken Charges and Frontier molecular orbitals (PDF)
