# Supplementary material for: Electronic and Optical Properties of Graphene Oxide Quantum Dots: DFT Insights of Mixed Vacancy and Dopants
Source: ACS Omega. 2026 Jun 22;11(26):38750–62. doi: 10.1021/acsomega.6c01895 (PMC13347341; doi:10.1021/acsomega.6c01895)

Electronic and Optical Properties of Graphene Oxide Quantum dots: DFT insights of mixed vacancy and dopants

Edna da Silva Machado[a]; Nailton Martins Rodrigues[b]; João Batista Lopes Martins[a]

[a] Instituto de Química, Universidade de Brasília, 70910-900 Brasília, DF, Brasil

[b] Departamento de Química, Universidade Federal do Maranhão, 65085-580 São Luís, MA, Brasil

Supporting Information

Figure S1. Schematic route for the generation of defective GOQDs. The bond lengths between the atoms involved in the defects (pore size, in Å) are emphasized.

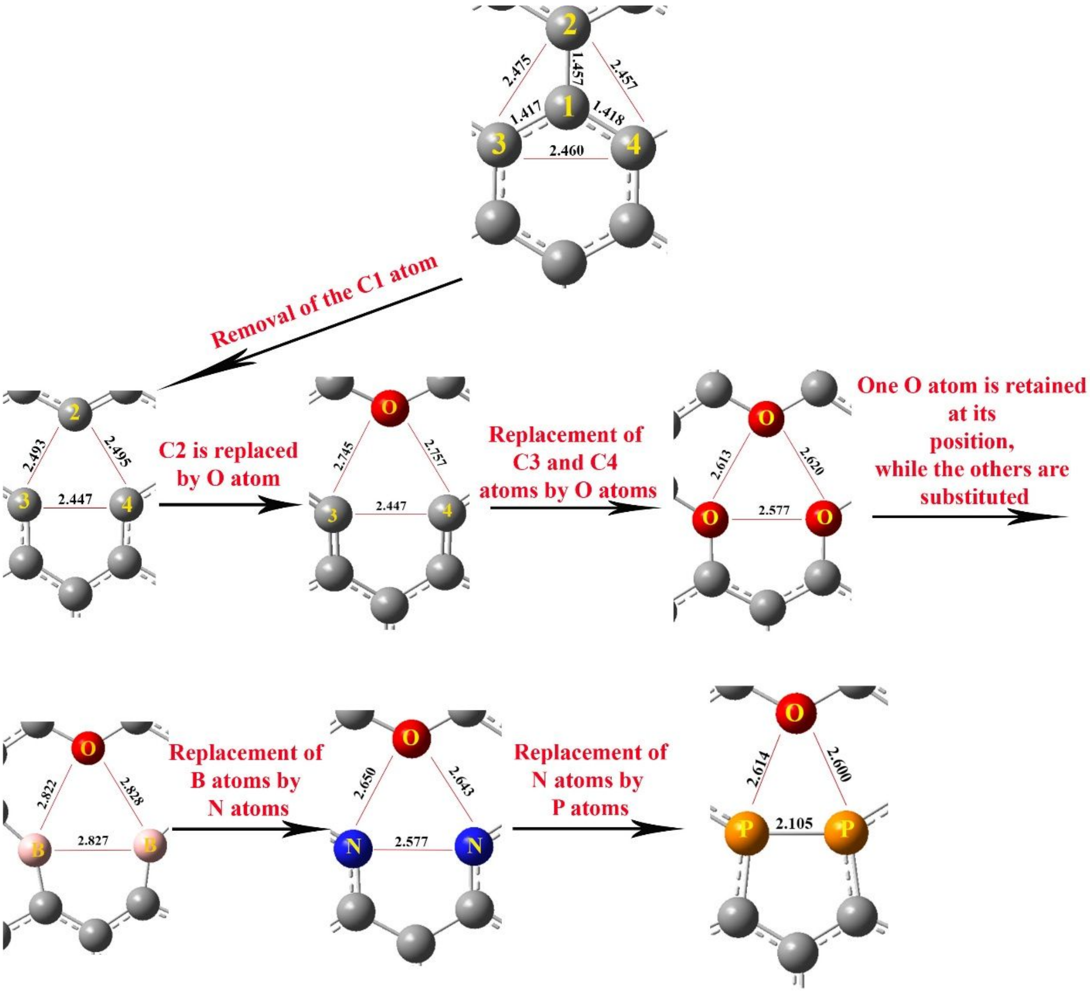

Table S1. Mulliken Charges of different GOQDs.

Atom		Pristine GOQD	SV-GOQD		O-SV-GOQD		O_3-SV-GOQD
1	C	0.3572436807	0.54781476	C	0.4669679601	C	0.6387990981
2	C	-0.1121144825	-0.25556900	C	-0.2392327308	C	-0.3310385608
3	C	0.0298665781	0.14378366	C	0.2529134167	C	-0.0336241696
4	C	-0.1328874154	-0.30101913	C	-0.2788502345	C	-0.4424576909
5	C	0.3748281410	0.57243466	C	0.4722036532	C	0.7692126917
6	C	-0.0235783665	-0.40633153	C	-0.3418023656	C	-0.2772126353
7	C	0.0557042967	-0.17066323	C	-0.3946603313	C	0.4748075977
8	C	0.2575196129	0.10487336	C	0.2242250371	C	0.1592165675
9	C	-0.1757935435	-0.01375541	C	-0.1168527392	C	0.0827749780
10	C	0.0978510151	-0.03378694	C	0.1665926870	C	-0.4676205446
11	C	-0.0578061926	-0.18520074	C	-0.1323151366	C	0.0384816681
12	C	0.2510803187	0.33088001	C	0.2603974562	C	0.2170226541
13	C	-0.2966127839	-0.35089603	C	-0.3436973397	C	-0.2959413848
14	C	0.0221996329	0.05283679	C	0.1025914239	C	0.0911960436
15	C	-0.0075475610	-0.01732606	C	0.0439924449	C	-0.1667987579
16	C	-0.5167299396	-0.69500330	C	-0.7321831282	C	-0.7568248718
17	H	0.1209074720	0.15361647	H	0.1477299951	H	0.1545697598
18	C	0.1388180314	0.00417290	C	0.1339513514	C	0.0373899212
19	C	-0.1380380130	0.05101120	C	-0.1630042401	C	0.1262959914
20	C	0.0920310525	-0.13158209	C	0.1677706225	C	-0.3584066045
21	C	0.0979261714	0.46774836	C	-0.0986020464	C	0.3001004792
22	C	0.0922899257	0.31050957	C	0.0104897906	C	-0.0904989297
23	C	-0.0128611525	0.16628920	C	-0.5599897940	C	-0.5137880409
24	C	-0.1382039004	-0.18888175	C	-0.6059210811	C	-0.6637965793
25	C	0.0299904056	-0.32174770	C	0.8968931212	C	0.7414857169
26	C	-0.1121562413	-0.31601877	C	0.4974634466	C	0.5326171755
27	C	-0.1330783481	-0.34702733	C	-0.4413864883	C	0.2557984650
28	C	0.3572399551	0.63049902	C	0.3303330199	C	0.0281627719
29	C	0.3746698711	0.59302792	C	-0.1538781723	C	-0.2728392149
30	C	-0.0237685654	-0.44551891	C	-0.3276868123	C	-0.1392436428
31	C	0.1389049350	-0.09094876	C	-0.7949629050	C	-0.7112430295
32	C	-0.0141035965	0.08359214	H	0.1091691325	H	0.1189191258
33	C	-0.0811614594	-0.06376464	C	0.0188436466	C	0.1424808991
34	C	-0.4070510697	-0.58736176	C	-0.2477853017	C	-0.2970372289
35	H	0.1059042104	0.11371335	C	-0.2183568080	C	-0.1870187702
36	C	0.0741464251	0.35995922	C	-0.1422193320	C	-0.2478752413
37	C	-0.1173977873	-0.24915114	C	0.5202421010	C	0.2000003389
38	C	-0.2283605939	-0.22060518	C	-0.1307338332	C	0.1087040646
39	C	-0.1758190939	-0.29511528	C	-0.0618025895	C	-0.1681171946
40	C	0.2576077662	0.00084476	C	0.1590707743	C	0.1351147241
41	C	-0.0577614469	0.04686072	C	-0.3645617135	C	-0.4520569403
42	C	0.0219934827	0.08849198	H	0.2104581176	H	0.2110096822
43	C	0.2510473298	0.29289180	C	-0.4941972558	C	-0.6336453438
44	C	-0.2961910785	-0.42057186	C	-0.0150255813	C	0.0420069343
45	H	0.1872964717	0.20419599	C	0.2011810733	C	-0.0339920391
46	C	-0.3004276387	-0.59657716	C	-0.2432889277	C	-0.1703422422
47	C	-0.0129985410	-0.05184778	C	-0.1150274438	C	-0.1342680749

48	C	-0.0139163295	0.01568852	C	0.1495996307	C	0.3596270765
49	C	-0.1171098821	-0.20874612	C	-0.6745951328	C	-0.4854886325
50	C	-0.0815625777	-0.09063775	H	0.1049584832	H	0.1184733998
51	C	0.0742986984	0.42126970	H	0.1292533887	H	0.1410065248
52	C	-0.4070396354	-0.57491599	C	-0.0552215760	C	0.0708244792
53	H	0.1058968159	0.11544051	C	-0.2844719072	C	-0.2147244220
54	H	0.1295233392	0.13455149	C	-0.7823653518	C	-0.8058288126
55	C	-0.0076786522	0.12418107	C	-0.5421178318	C	-0.5649428785
56	C	-0.2285442836	-0.23772654	H	0.1566951350	H	0.1617647869
57	C	-0.5165825581	-0.70486133	H	0.1364215223	H	0.1414260515
58	C	-0.2996586246	-0.62741549	H	0.1966061677	H	0.2118212871
59	H	0.1209082939	0.15394265	H	0.1334284819	H	0.1368332651
60	H	0.1387939736	0.14235589	H	0.1390574756	H	0.1431569623
61	H	0.1874196843	0.20773128	C	0.8131944883	C	0.7420554659
62	H	0.1295298888	0.13130262	O	-0.3113863223	O	-0.3035371965
63	H	0.1388233946	0.14332166	O	-0.2416011952	O	-0.2702738447
64	C	-0.1128465002	0.79790900	H	0.3165665436	H	0.3177497977
65	O	-0.2251487024	-0.31515074	C	1.5055586809	C	1.3114633840
66	O	-0.1257045986	-0.27098455	O	-0.3065222770	O	-0.3330836627
67	H	0.2593880326	0.31432312	O	-0.2350330894	O	-0.2746612043
68	C	-0.1128009274	0.93202325	H	0.3198537569	H	0.3128861556
69	O	-0.2251605545	-0.29035805	C	0.5649434800	C	0.6325836073
70	O	-0.1257141669	-0.25266884	O	-0.3010096334	O	-0.3082737257
71	H	0.2593893256	0.31985694	O	-0.2121943764	O	-0.2395029801
72	C	0.5100827318	0.63432576	H	0.2872417701	H	0.2901107185
73	O	-0.2911164985	-0.29514900	C	0.6170321073	C	0.5975567820
74	O	-0.2113087847	-0.23463310	O	-0.3084483221	O	-0.2930367783
75	H	0.2844363087	0.29196730	O	-0.2140480833	O	-0.2405203694
76	C	0.5091496496	0.66608026	H	0.2880798556	H	0.2928241322
77	O	-0.2908850823	-0.29979411	O	-0.1923524712	O	-0.2069413662
78	O	-0.2111937019	-0.23916845	H	0.2638157197	H	0.2610040458
79	H	0.2843802723	0.29146562	O	-0.2003749088	O	-0.1958663081
80	O	-0.2033076758	-0.18776273	H	0.2618080675	H	0.2624940214
81	H	0.2600427792	0.26276171	O	-0.2112286943	O	-0.2093078954
82	O	-0.2032815707	-0.20177724	H	0.2587654068	H	0.2613593038
83	H	0.2600432453	0.26170131	O	-0.2028979305	O	-0.2101696334
84	O	-0.2144735000	-0.21032646	H	0.2597095864	H	0.2581839340
85	H	0.2586914057	0.25784184	O	0.2367359898	O	0.1095956064
86	O	-0.2144755218	-0.20035448	C	0.2607006879	O	0.1409840010
87	H	0.2586913590	0.25861304	C	0.2403867026	O	0.1198953169
88	C	0.0554032163					

*The atoms highlighted in red are heteroatoms.

Table S2. Mulliken Charges of different GOQDs.

Number	O-B_2-SV-GOQD		O-N_2-SV-GOQD		O-P_2-SV-GOQD	
1	C	0.5300534225	C	0.7750946308	C	0.4910498867
2	C	-0.1770745897	C	-0.3602953443	C	-0.2039826743
3	C	-0.1611985388	C	0.1282879915	C	-0.1648153564
4	C	-0.2408067305	C	-0.4293432164	C	-0.2735249164
5	C	0.5453803405	C	0.7912265330	C	0.5415895830
6	C	-0.2222819788	C	-0.2365400786	C	-0.1838916740
7	C	0.5228262772	C	0.3643081960	C	0.4179018412
8	C	0.2186915025	C	0.1877012064	C	0.2454144805
9	C	-0.1067903863	C	0.2314486810	C	-0.3044564691
10	C	-0.2851873264	C	-0.6143466941	C	-0.4554185819
11	C	0.0134105864	C	-0.0276079772	C	0.1025864902
12	C	0.1885853566	C	0.1239397182	C	0.0593810064
13	C	-0.3084301362	C	-0.2029489565	C	-0.3296567641
14	C	0.0845577283	C	0.1978244464	C	0.2510405127
15	C	-0.1240442675	C	-0.2485244686	C	0.0772892710
16	C	-0.6418431540	C	-0.8611987501	C	-0.7850744087
17	H	0.1459920378	H	0.1463704789	H	0.1505556048
18	C	0.1335333558	C	0.0767494762	C	0.1907237247
19	C	-0.0710162934	C	0.2452173081	C	-0.2167895723
20	C	-0.2510599977	C	-0.5133063663	C	-0.4621727597
21	C	0.0248757063	C	0.3102377295	C	-0.2043860515
22	C	-0.0956807306	C	-0.3232801961	C	0.0619501816
23	C	-0.5698659084	C	-0.7057397859	C	-0.7441082568
24	C	-0.6684503881	C	-0.8114570601	C	-0.7537380093
25	C	0.7937238602	C	0.8051812294	C	0.8739279599
26	C	0.5033415155	C	0.5400929380	C	0.5173492362
27	C	0.2162875978	C	0.2907845940	C	0.0048788464
28	C	0.1577219533	C	0.2108015437	C	0.3055955947
29	C	-0.0261609356	C	-0.2145139389	C	0.1016530310
30	C	-0.3080154576	C	-0.2699939258	C	-0.3292521892
31	C	-0.8234318099	C	-0.7296829895	C	-0.8689728263
32	H	0.1131841434	H	0.1100692480	H	0.1176399511
33	C	0.1866908393	C	0.0598968543	C	0.2160750232
34	C	-0.2011356720	C	-0.2579113535	C	-0.0569886306
35	C	-0.2236442879	C	-0.1920225107	C	-0.3576355614
36	C	-0.1798334835	C	-0.4499809525	C	0.1837287395
37	C	0.2812662790	C	0.3303404845	C	0.2850316586
38	C	-0.0508754001	C	0.1039355090	C	-0.5221718313
39	C	-0.0897849853	C	-0.0717992703	C	-0.0939285697
40	C	0.2831643692	C	0.1717833853	C	0.3944330550
41	C	-0.3450255145	C	-0.4214142546	C	-0.3468504858
42	H	0.2081406428	H	0.2046300900	H	0.2089741481
43	C	-0.5091835764	C	-0.5967450826	C	-0.6570797589
44	C	0.0476843325	C	-0.0255803255	C	0.2641778358
45	C	0.0920458837	C	0.1152671478	C	0.1921744075
46	C	-0.2588039121	C	-0.1576171306	C	-0.1866645027
47	C	-0.1865588180	C	-0.2813843477	C	-0.1452070241

48	C	0.0696514544	C	0.1747699494	C	-0.0742850904
49	C	-0.5835157951	C	-0.7016462953	C	-0.6187252107
50	H	0.1053950206	H	0.1035851797	H	0.1086160510
51	H	0.1298666615	H	0.1309296733	H	0.1347687332
52	C	0.0443888628	C	-0.0748382052	C	0.0577450881
53	C	-0.2340033784	C	-0.1719993768	C	-0.3626382071
54	C	-0.7833931234	C	-0.7905156198	C	-0.8693694401
55	C	-0.4567015942	C	-0.5469871089	C	-0.5966900086
56	H	0.1597169667	H	0.1549979226	H	0.1668902671
57	H	0.1377873213	H	0.1365639023	H	0.1422324830
58	H	0.1951811850	H	0.1964728287	H	0.2023323292
59	H	0.1383072139	H	0.1319743247	H	0.1392427839
60	H	0.1391045319	H	0.1394818892	H	0.1408589130
61	C	0.7272925298	C	0.7665513706	C	0.7534326061
62	O	-0.3069162381	O	-0.3083497093	O	-0.3148188965
63	O	-0.2402521886	O	-0.2431339957	O	-0.2440884684
64	H	0.3173423318	H	0.3178398228	H	0.3160686143
65	C	1.3282296673	C	1.1934986606	C	1.6083365653
66	O	-0.3224550157	O	-0.2987312603	O	-0.3628723452
67	O	-0.2486058435	O	-0.2363330828	O	-0.2680550549
68	H	0.3171288540	H	0.3212577515	H	0.3102171138
69	C	0.5291777678	C	0.5718266705	C	0.8776053347
70	O	-0.3014283718	O	-0.3002867055	O	-0.3097600008
71	O	-0.2153375412	O	-0.2111309941	O	-0.2210149891
72	H	0.2871156781	H	0.2871679548	H	0.2877163636
73	C	0.5727898608	C	0.5589393762	C	0.9452793055
74	O	-0.3038848648	O	-0.3071663509	O	-0.2963649318
75	O	-0.2149658885	O	-0.2130455560	O	-0.2161097292
76	H	0.2880700362	H	0.2868718399	H	0.2900962345
77	O	-0.2082676280	O	-0.1884863118	O	-0.2175449658
78	H	0.2635452234	H	0.2627205328	H	0.2639021210
79	O	-0.1974461842	O	-0.2028848247	O	-0.1925689497
80	H	0.2621100112	H	0.2620145639	H	0.2635118636
81	O	-0.2103930545	O	-0.2153964606	O	-0.2068066839
82	H	0.2590001333	H	0.2596916288	H	0.2609957042
83	O	-0.2156838220	O	-0.1981125994	O	-0.2224917475
84	H	0.2598384396	H	0.2590940320	H	0.2610180573
85	O	-0.0004930484	O	0.3553853492	O	0.0138225762
86	B	0.1722388692	N	0.4170135023	P	0.2679355632
87	B	0.1754914664	N	0.4024412331	P	0.1732248679

* The atoms highlighted in red are heteroatoms.

Figure S2. Frontier molecular orbitals and their respective HOMO-LUMO energy gaps (E_g).

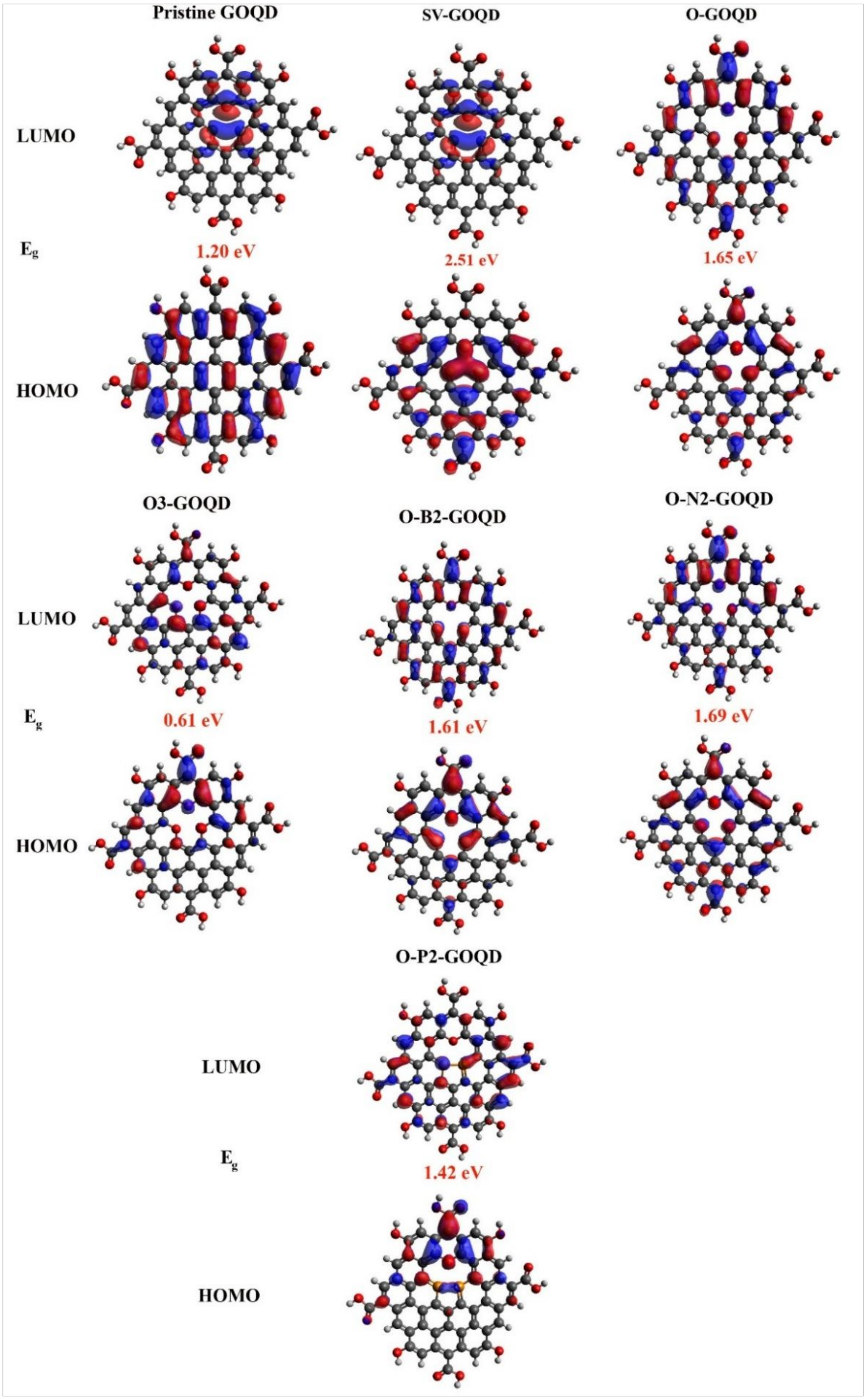

Figure S3: Molecular orbitals involved in electronic transitions for the of the a) pristine GOQD, b) SV-GOQD, c) O-SV-GOQD, d) O3-SV-GOQD, e) O-B2-SV-GOQD, f) O-N2-SV-GOQD, and g) O-P2-SV-GOQD.

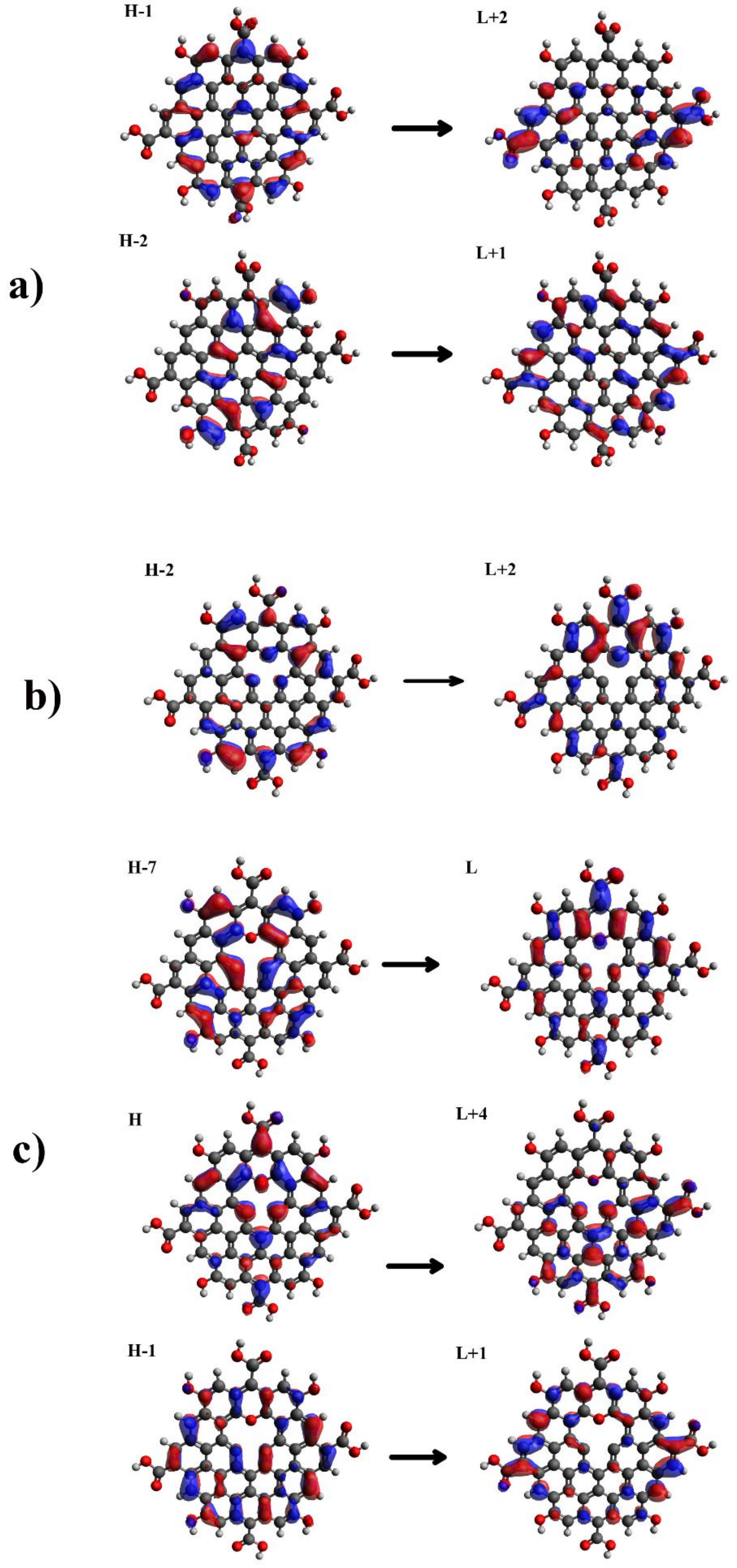

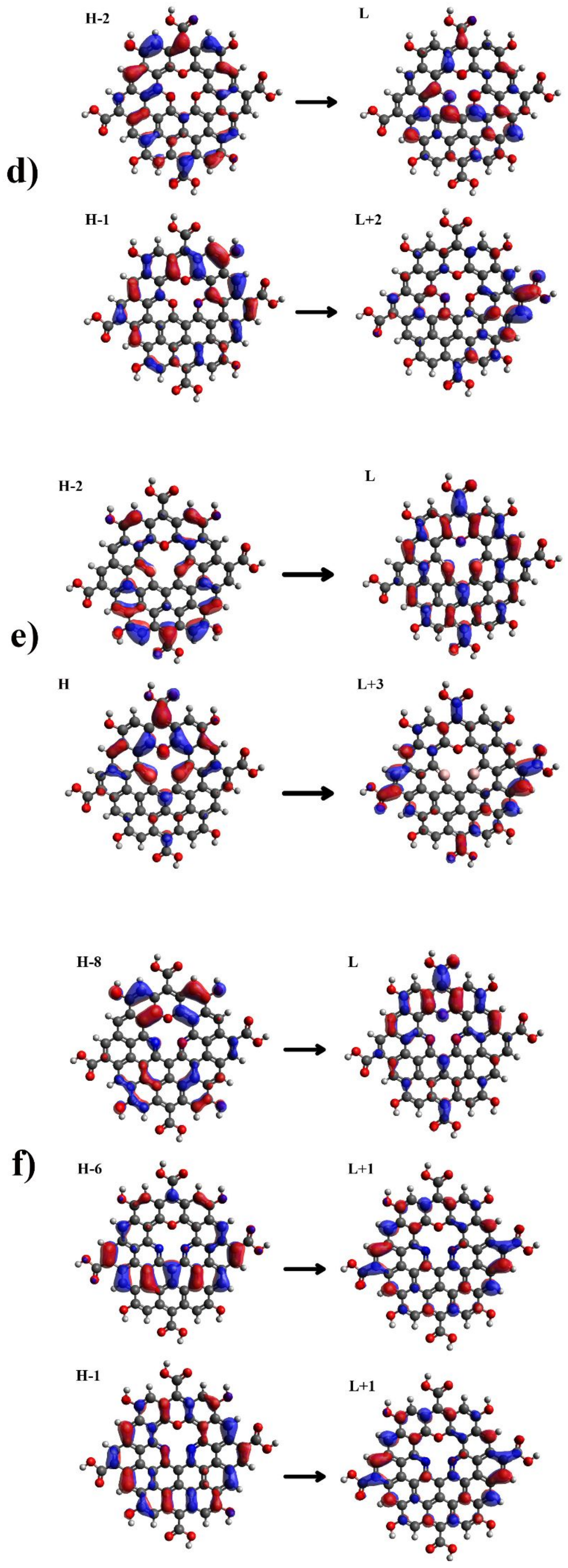
d)
H-2
L
H-1
L+2
e)
H-2
L
H
L+3
f)
H-8
L
H-6
L+1
H-1
L+1

g)

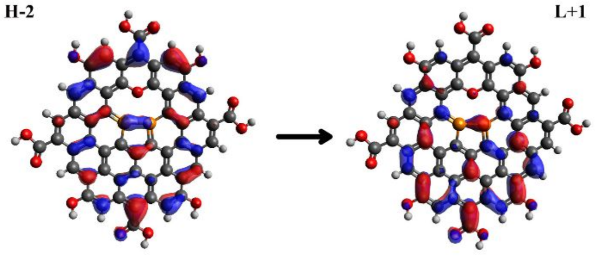

Figure S4. Contribution (%MO) of atomic orbitals in the formation of the molecular orbitals involved in the electronic transitions of the a) pristine GOQD, b) SV-GOQD, c) O-SV-GOQD, d) O_3-SV-GOQD, e) O-B_2-SV-GOQD, f) O-N_2-SV-GOQD, and g) O-P_2-SV-GOQD. The bar colors represent contributions from different atomic orbitals. C(2s), blue to O(2s), red to the O(2p) orbitals of the heteroatom, violet to B(2p), orange to N(2p), and green to the d orbitals. Only orbitals with relatively small contributions are included for clarity.

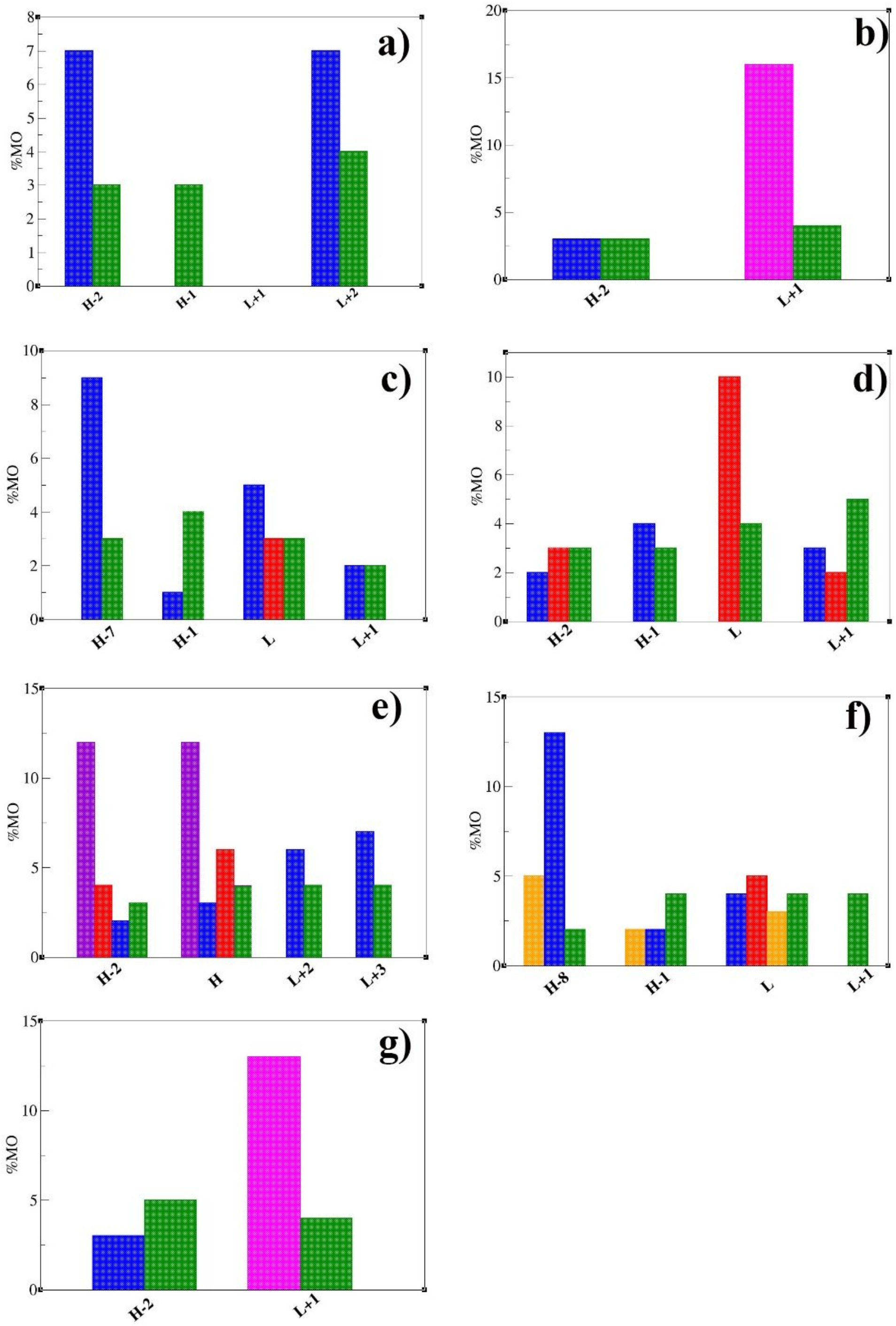

Supplement: Supplementary file 1 [file ao6c01895_si_001.pdf]
